# An Attempt to Explain Visual Aesthetic Appreciation

**DOI:** 10.1007/s12124-022-09701-8

**Published:** 2022-05-18

**Authors:** Bjørn Grinde, Tammy-Ann Husselman

**Affiliations:** 1 Division of Mental and Physical Health, Norwegian Institute of Public Health, Oslo, Norway; 2 University of Central Lancashire, Preston, UK

**Keywords:** Aesthetics, Visual stimuli, Evolution, Rewards and punishments, Supernormal stimuli

## Abstract

We suggest an evolutionary based explanation for why humans are preoccupied with aesthetic aspects of visual input. Briefly, humans evolved to be swayed by positive and negative feelings in the form of rewards and punishments, and to pursue situations that induce rewards, even when the feeling is not sufficiently strong to be recognized as a reward. The brain is designed to offer rewards when a person focuses on certain types of visual stimuli. For example, warm colors are typically pleasant because they are associated with edible fruits, and complex images appeal to curiosity. At some point people began exploiting these types of brain rewards by beautifying objects and creating art. The utility of objects, and the associative (or communicative) aspects of art, may dominate the design, but the artist tends to add aesthetic elements. These elements imply visual aspects that do not add to the functional value or evoke memories or associations based on easily recognized features in the picture. The adaptive rationale for the rewards offered by the aesthetic elements should help explain human aesthetic appreciation.

## Introduction

Hominins gradually evolved to walk in an upright position, thereby freeing their hands for the manipulation of tools. There is no doubt that the ability to use tools was of great importance for the success of humankind. It is less obvious why our forebears started to add beauty to their tools in ways that did not enhance utility but were widely recognized as pleasing. Eventually, humans moved one step further in that we create objects of art with no obvious practical value. The artist may claim that the work is meant as a way of communicating, but the point remains that aesthetic qualities are added that do not appear to be required for this purpose. Here we focus on these aesthetic qualities rather than the function of art in facilitating communication and creating associations.

Aesthetics has been described as “the study of how and why sensory stimuli acquire hedonic value” (Skov & Nadal, 2020) – which means it is a question of features of visual stimuli, here referred to as *aesthetic elements*, that for some reason activate reward circuits in the brain. *Aesthetic behavior* is then allotting time and energy to the production of aesthetic artifacts, or viewing such artifacts, for the sake of the rewards offered. Some form of aesthetic behavior seems to be present in practically all cultures examined by anthropologists (Benton & DiYanni, 2012), strongly suggesting that the behavior is rooted in innate tendencies. As it appears to reflect a universal human attribute, the reward mechanisms presumably evolved in accordance with stimuli coming from nature; that is, directing attention to these types of sensory input was adaptive in our evolutionary past.

Today the individual does not necessarily obtain any adaptive benefit from aesthetic behavior, yet an evolutionary perspective can help explain the phenomenon. Several texts have used an evolutionary perspective to explain aesthetics (Davies, 2012; Grinde, 1996; Kozbelt, 2017; Rolls, 2017; Westphal-Fitch & Fitch, 2018), we believe the present approach offers novel insight. The approach is in line with what has been described as the hedonic school of aesthetics; specifically, the aesthetic value is a question of pleasing the viewer (Matthen, 2017). We are aware of critics of this school (Van der Berg, 2020), but believe the main objections to the hedonic approach are considered by the following two constraints. For one, we take advantage of a novel understanding of how the *mood modules*, the brain circuits that form rewards and punishments, function; in other words, the term pleasure is given a somewhat broader meaning (Grinde, 2016). Two, we restrict what is included in the term ‘aesthetic behavior’ – as will be discussed below.

## Aesthetic Behavior

The rewards of viewing aesthetic elements can be obtained from nature, thus spending time staring at a ripe strawberry or a sunset can be construed as aesthetic behavior. However, we shall limit the scope of this text to manmade objects.

There is one further constraint, we are interested in the subtle aesthetic qualities that are present regardless of the communicative or representative content. This constraint excludes at least some of the pleasure of seeing a nice landscape or a friendly face, but the restriction is somewhat vague. If artists want to communicate joy, they may use bona-fide aesthetic elements; and figurative content has some bearing on how our capacity to enjoy aesthetics was formed. Yet, we believe there are core aesthetic elements that work in the absence of other subject matter. In short, although art can induce a range of emotions and feelings by various mechanisms (Menninghaus et al., 2019; Schindler et al., 2017; Tinio & Gartus, 2018), we are interested in the intuitive pleasures of beauty. The constraint also excludes other reasons for taking an interest in aesthetic objects, such as the potential for obtaining high status in society and the monetary value of the objects.

The above delineation reflects that aesthetic behavior is necessarily mixed in with other aspects of human behavior. There is a parallel in religious activities, they are not solely about connecting with supernatural entities, but includes, for example, socializing as well as aesthetic behavior in the form of religious art.

It has been reasonably well documented that art can induce pleasure (Lacey et al., 2011; Skov, 2010; Vartanian, 2018), and we assume that some of the pleasure derives from aesthetic elements that remain after the above restrictions. These elements may be more obvious in the design of utensils, such as vases or houses, compared to in actual works of art (because the figurative or associative content is more important in art), but the principles guiding the potter also apply to the painter. Aesthetic intention is reflected in features included to make an object more visually attractive. In the case of paintings, the importance of the figurative content implies that the present text focuses on what may be a minor aspect of the appreciation of art: Our inborn tendency to enjoy specific types of visual input regardless of the context they are in. It follows that a painting can be pleasurable to look at in the absence of any aesthetic qualities; for example, no artistry is needed to make a rendering of nude women that delights a man.

We shall start by outlining a model of how the brain uses rewards and punishments to motivate behavior. Based on this model, we consider whether aesthetic behavior occurs in other species, and (if not) at what point it emerged in the hominins. Subsequently, we suggest various features of visual stimuli that are likely to be involved in aesthetics.

## The Brain’s Mood Modules

### Rewards and Punishments

The term *feelings* as used here includes visual (and other) sensations that have an element of something either positive or negative; that is, the sensation activates *rewards* or *punishments* in the brain. The dual nature of feelings goes back to their role of instigating behavior – the main options for any animal are to either move ‘towards’ what benefits the genes (rewards) or ‘away from’ whatever is detrimental (punishments) (Grinde, 2016).

The various functions evolution has added to the brain can, for convenience, be referred to as *modules* (Friston & Price, 2011; Grinde, 2012). The term module does not imply a particular location in the brain, the relevant neurological circuits can be widespread and overlapping with other modules.

The conscious experience of feelings is based on activity in two different sets of modules: the *mood modules* and the *type modules*. The positive or negative feelings are due to the contribution of what we refer to as mood modules. They were presumably introduced at the amniotic stage of vertebrate evolution as a strategy for creating a ‘common currency’ when evaluating various options of behavior (Cabanac, 1999; Grinde, 2018). In other words, the strength of expected rewards is weighed up against potential punishments. The mood modules are typically activated by another set of modules, which are here referred to as type modules; the module responsible for processing visual input (situated in the visual cortex) is one example. Input from mood and type modules merge in a moment of conscious experience. The type modules add the specific quality to the experience; they explain why, for example, the pleasure of viewing a beautiful flower feels so different from hearing a favorite song.

Based on neurological research, the mammalian brain has three distinct mood modules (Berridge & Kringelbach, 2015; Leknes & Tracey, 2008). Negative feelings originate from a single *pain module*, which is why all sorts of negative feelings, whether in the form of mental agony or physical pain, can be included in the term punishments. The reward system is divided into two modules, the *seeking module* (also referred to as wanting or motivating) is meant to stimulate the individual to seek opportunities and motivate for relevant action; the *liking module* (also referred to as consuming) makes sure the opportunities are exploited. The effect of the seeking and liking modules can be exemplified by a person who first follows the smell of a bakery and then eats a cake.

The classification of the two reward modules is based on their neurobiology but also makes sense in evolutionary terms (Grinde, 2012). That is, to obtain what is good for the genes, it is necessary to first motivate the individual for action and then ensure that the opportunity ends with actual consumption. A single pain module suffices for whatever the individual should avoid. In short, the same neurological circuits apparently create the positive or negative content regardless of the type of situation or stimuli (Blood & Zatorre, 2001; Leknes & Tracey, 2008; Lieberman & Eisenberger, 2009). For simplicity, input from the two reward modules is combined in the term rewards. The main ‘switches’ for turning the mood modules on or off belong to the unconscious part of the brain, for the obvious reason that they are meant to control you, rather than you control them.

In this model of the mind, the type modules activate the mood modules (Grinde, 2012). The latter solely adds the positive or negative component of the experience. It is relevant to note that a particular type module can activate either pleasure or pain. Fear is a typical example, normally it activates the pain module, but a fearful situation can also offer pleasure, as in the case of the adrenalin kick of a climber. Even grief sometimes feels good (O’Connor et al., 2008). Both rewards and punishment can be activated simultaneously, or the brain can switch rapidly from one to the other; in the case of the climber, the kick will change to panic if he slips.

Sensory input may elicit both rewards and punishments. Their combined effect determines how the moment is experienced. For example, the pleasantness of an odor such as jasmine can be enhanced if it is combined with a component such as indole that has an unpleasant smell (Rolls, 2017). The enhancement may be due to the contrast provided by the unpleasant part, or because this part heightens the attention to odors in general. Presumably, similar principles can be at work in the case of visual stimuli.

It should be noted that the rewards and punishments are not necessarily recognized consciously. Small ‘drops’ of either pleasure or pain can be acted on even if the person is unaware of why the action, or the interest, is instigated (Tamietto & de Gelder, 2010). It follows that the aesthetic elements discussed below, may or may not be recognized as such by the viewer.

One line of investigation has been to look for the neurological correlates of an aesthetic experience, a field sometimes referred to as *neuroaesthetics* and based primarily on brain imaging studies (Cela-Conde & Ayala, 2018; Chatterjee & Vartanian, 2014). The brain must necessarily activate structures associated with visual processing (in visual arts), attention, and rewards. Moreover, the circuits required for the creation of a conscious experience, which is sometimes referred to as ‘broadcasting’ and include the corticothalamic complex, are active (Dehaene et al., 2014). The brain features mentioned in this paragraph as required seem to correlate reasonably well with the reported observations as to regions of the brain active while engaged in aesthetics – given the uncertainty of brain imaging – thus in our opinion it is not necessary to postulate specific brain circuits (or modules) dedicated to aesthetics. The compelling question is why certain types of visual stimuli activate the reward system.

### Supernormal Stmuli

Time spent on aesthetic behavior does not need to be adaptive in the sense that it contributes to survival or procreation, the behavior can be explained by the nature of the reward system in the brain. Paying attention to certain types of visual input when out in nature is (or was) presumably adaptive, but this tenet only forms a basis for aesthetic behavior, the substantial allocation of time and resources can be explained by the concept of *supernormal stimuli*.

Supernormal stimuli imply something that elicits a stronger response than the natural stimuli for which the response evolved (De Block & Du Laing, 2010). Chocolate is an example. The taste of both sugar and fat offers rewards, the producers have fine-tuned their chocolate recipes for maximal effect (Casperson et al., 2019). Pornography can be construed as another example as it offers sexual stimuli that tend to be superior to what a person is likely to experience in real life. Our propensity for eating chocolate and masturbating to pornography is not adaptive behavior, but a consequence of opportunities available in the present environment. Aesthetic behavior is somewhat related, but only in that the artists tend to exaggerate the effect of the natural stimuli for which the visual rewards evolved. Other species of birds and mammals display similar behavior in that they prefer to engage with supernormal stimuli (Barrett, 2010), presumably because these offer stronger rewards compared to natural stimuli and because the animals adhere to the principle of choosing behavior that maximizes pleasure.

Engaging in supernormal stimuli may be biologically unproductive, in that the time spent is wasted or the consequences are detrimental to health, but it is not necessarily maladaptive in terms of happiness. We believe the pertinent question to be how the behavior affects (long-term) quality of life. Although an artist’s preoccupation with art can in some cases have a negative effect on health, in that the behavior becomes so all-consuming that basic sustenance is not cared for, most people are likely to improve their lifetime sum of happiness by engaging in aesthetics.

It is possible that the recent evolution of our lineage has been in the direction of enhancing our interest in art. If those with artistic talent were preferred as partners, sexual selection would help explain why the production of aesthetic objects is such a prominent feature of society (Prum, 2012). There are, however, reasons to assume that sexual selection has limited relevance. For one, the present human populations started to split more than 150,000 years ago (Schlebusch et al., 2017). It is not obvious that our ancestors spent much time on aesthetic behavior prior to that. If aesthetic behavior is a consequence of sexual selection occurring after that time, it would imply either convergent evolution or that the behavior is present only in particular populations. Furthermore, sexual selection generally takes as a starting point features, or behaviors, that are already present. Thus, the consequence of sexual selection would be to strengthen the engagement in aesthetic behavior, but this form of selection is unlikely to explain why the behavior appeared in the first place.

## Aesthetic Behavior in Animals and Early Humans

All mammals have brain structures corresponding to those responsible for the mood modules in humans. Moreover, some of the features of visual input that can activate rewards are presumably relevant for at least our closer relatives such as the apes; for example, fruit is typically included in their diet thus they should take an interest in warm colors. In short, at least the apes presumably have the cognitive requirements for aesthetic appreciation, but actual behavior also depends on opportunity. In humans, opportunity is cared for by the dexterity of our hands and the intellectual capacity to produce the means to engage in artistic processes.

Apes have some dexterity, but normally lack the means. If given the chance, at least some of them do engage in aesthetic behavior. Desmond Morris describes the chimpanzee Congo who became fond of painting pictures (Morris, 1994). Congo did not produce figurative images, but he did show an appreciation for color, and he preferred a balanced, patterned composition with geometrical shapes such as fans and circles. He was not rewarded for participating, and he protested if anyone tried to remove him before he considered a painting finished, which strongly suggests aesthetic behavior in the present sense.

The point is substantiated by the observation that human children behave in a similar way. By 2–3 years of age, their dexterity is sufficient to use a crayon, but the first images are non-figurative scribble (Morris, 1994). By the age of 4–5, they start to depict geometrical shapes and subsequently pictorial images. The behavior is performed voluntarily and is pretty much the same regardless of the culture the children belong to; only later does their ‘art’ reflect their upbringing. As in the case of Congo, they enjoy the process, but do not care much about the product.

The aesthetic behavior of children and of Congo can be described as a form of play behavior. Play evolved for infants to learn skills required as adult, for example in the form of controlling hand movements and learning to recognize and interpret visual input. In most species, play behavior ceases when the individual approaches adulthood, but in humans it seems to linger, perhaps because we are in greater need of learning throughout our lives. The point presumably helps explain the prevalence of aesthetic behavior in adults. Then again, while all children enjoy their crayons, modern human societies generally move in the direction of specialist behavior, whether in art or engineering, which means that aesthetic production tends to be primarily in the hands of professional artists.

Certain aspects of animal behavior have an aesthetics component, for example, peacocks showing off their colorful plumage, the nest building of bowerbirds (Borgia, 1986), and the presentation of nuptial gifts (Lewis & South, 2012). However, it is not obvious that these examples qualify as aesthetic behavior according to the present demarcation. Decorative bodies are generally not something the animals create, but simply a product of sexual selection. Nests and nuptial gifts are created (at least collected) by the animals, and the process may involve pleasure for both the creator and the spectator, but the behavior is part of courtship, and the animals seem unlikely to spend time on similar behavior in the absence of a chance for mating. In short, animals in the wild do not appear to produce visual artifacts simply for the joy of the creation, their delight is more akin to what humans’ experience when finding a ripe fruit or spotting a potential lover. Humans find aesthetic value in the peacock’s tail, but for the bird it is solely a feature tuned to species-specific requirements for procreation.

Based on the above discussion, true aesthetic behavior is likely to be rare outside the human lineage; however, it may predate *Homo sapiens*. Some three million years ago one of our Australopithecine ancestors found a pebble with a clear resemblance of a face and decided to bring it back to his or her cave. Actual production of artifacts with apparent aesthetic and/or symbolic intent date back 300,000 years or more (Kissel, 2018). When considering aesthetic activity in our distant ancestors, it is important to keep in mind that only objects made of a hard material (such as rocks, shells, and ivory) are likely to survive. It is possible that early hominins spent time on aesthetic pursuits, for example in the form of woodcarving or drawing on hides with charcoal, even if no such remains are found. Yet, this form of behavior probably was of limited importance until the invention of more suitable means for creating aesthetic objects. Remaining artifact, in the form of cave paintings and figurines, are generally less than 50,000 years old (Clottes, 2016).

## Aesthetics Elements

### Color

Color preferences in humans depend largely on the choice of objects and the circumstances in which they are viewed (Gong et al., 2017; Torres et al., 2020). However, there is a general agreement that colors can enhance visual comfort (Birren, 2016; Elliot & Maier, 2014). Moreover, certain colors tend to have connotations that make them agreeable or disagreeable, if not under any circumstances, so at least in some situations.

Fruits (and berries) are meant to be eaten by animals, which is why they typically have a color that makes them conspicuous in the green environment of the forest – such as red, orange, or yellow. Humans seek and eat fruit, which is one reason why we have trichromatic color vision. The brain presumably offers a reward for paying attention to objects of relevant color so that we are more likely to spot the fruit and thus find a meal. This notion may explain a delight in warm colors (Palmer & Schloss, 2010). The fact that the early pigments found to be associated with human decoration are yellow and red, in the form of ochre, adds evidence to the idea of a preference in this direction. Apparently, ochre was used already by the Neanderthals, and the use dates back more than 200,000 years (Hodgskiss, 2020). Interestingly, Congo’s favorite color was red (Morris, 1994).

Red may be appreciated for another reason as well. The female genitalia are red or pinkish. In certain primates the buttocks of the female swell and turn pink when she is in heat, a signal employed to attract males (Dunbar, 2001). Thus, males may value the conscious or unconscious sexual associations accompanying something red, while females may value the color for its ability to attract men. The point is presumably reflected in the inclination to use red pigments in lipsticks and powder, as well as in the use of red in red-light districts. On the other hand, red is also associated with blood, and in industrialized cultures with alarm and the stop signal, associations that are more likely to cause negative feelings.

Humans tend to prefer a view of nature, compared to looking at a cityscape or an artificial environment, a tendency referred to as biophilia (Grinde & Patil, 2009). Beyond the chance of spotting edible objects, it seems reasonable that we can take delight in scenery suggesting a human-friendly habitat; that is, a lush place with water and options for cover (Brielmann & Pelli, 2018). One would expect this to imply an appreciation for green and blue. According to one study, women have a stronger preference for warmer colors, while men take comparatively more delight in blue-green (Hurlbert & Ling, 2007). The difference could be that women in the Stone Age were more focused on collecting fruit while men were more tuned to the general quality of the environment.

Brown tends to be undesirable as it is the color of feces and rotten fruit, both of which should be avoided (Palmer & Schloss, 2010). Most nuts come in shades of brown or gray. They are nutritious, but in contrast to fruit, not designed by the plant for animal consumption. The color of nuts may be partly intended as camouflage but may also reflect a strategy to make them less desirable for hungry animals.

Humans appear to prefer colors that are saturated and bright (Camgöz et al., 2002). Colors that are light and easily distinguished tend to be regarded as pleasant, while darker and muddier colors are more depressing. The effect of brightness may reflect that we are diurnal animals. Daylight is more useful for our activities than the dimness of the evening or early morning; we therefore thrive where there is plenty of light and tend to become depressed in its absence (Pjrek et al., 2020). Colors that are difficult to discern may leave us uncertain.

### Complexity and Fluency

Humans are pushed toward exploratory behavior, as are many other animals, because that way we may discover novel opportunities (Xu et al., 2021). In fact, one of the two reward modules is referred to as the *seeking system* (Wright & Panksepp, 2012). We enjoy gathering information because our curiosity can activate brain rewards. Our eyes are the most important tool for obtaining information regarding our surroundings. We cherish inspecting objects and scenery just for the sake of finding out more about the environment.

The term boring is used in aesthetics to describe visual input without sufficient variety or novelty. A painting may lack the required complexity in color or form to attract our attention; there are no surprising details to feed our curiosity and not enough content to make a visual scrutiny worthwhile. A suburb consisting of a single type of housing regularly spread out is considered depressing. On the other hand, originality, and variety – richness of detail and novelty – stimulate our curiosity and are therefore pleasing. In short, aesthetic liking is positively influenced if the design is complex (Landwehr et al., 2011) and novel (Hekkert et al., 2003).

Then again, we may lose interest or react negatively if the object or picture is too intricate (Papadimitriou, 2020). The mind needs clues to help it organize and understand visual input. There should be a degree of coherence in the diversity, as we need to find patterns or connections to make sense of the information presented. The confusion of not understanding an image is uncomfortable because strange or unfamiliar objects and settings could imply danger. Moreover, a heavy load of signals and impulses may lead to an unpleasant ‘overload’ in the conscious processing machinery (Lipowski, 1974). We are rewarded for understanding our environment and punished when we do not; that is, there appears to be an ‘inverted U-shape’ to the relationship between complexity and the pleasure obtained (Berlyne, 1970).

Conceptual fluency is a related term, it refers to how easy it is for the viewer to process the visual impact (Friedenberg & Liby, 2016; Graf & Landwehr, 2017). A painting should offer a spontaneous appreciation without the viewer having to struggle with disturbing visual elements. Too much complexity, or elements that do not ‘fit in,’ will tend to reduce the fluency. In order to grab our attention, the painting (or object) needs to induce an immediate positive experience.

Our visual system seems to be tuned to look for certain forms, or elements, that it can recognize – for example, basic shapes such as squares or circles – identifying these elements presumably offers drops of satisfaction. The notion could help explain the popularity of Cubism as a school of art, but the principle applies to any form of design, and is related to the above suggestion that it pleases us to find comprehensible information. As pointed out above, both human children and the chimpanzee Congo seem to appreciate geometrical shapes. Curvature is apparently better than straight and angular lines (Bar & Neta, 2006), perhaps because curves offer more in terms of complexity.

### Symmetry and Balance

One of the few rules of aesthetics that has won a certain acceptance is the *golden ratio* (a proportion of 1:0.62) (Konecni, 2003). For example, the main item in a painting should divide the canvas in this proportion. What the principle possibly suggests is that a 1:1 ratio is boring, while a too skewed ratio produces an unbalanced appearance. The golden ratio is then the optimal compromise between these two undesirable extremes.

The disagreeable effect of an unbalanced picture may relate to our fear of falling. Falling is not a major problem for an animal based on the ground, but our ancestors did once live in trees. Moreover, even ground-dwelling animals need to be aware of the possibility of an unsteady rock tipping over and making them stumble. A dominant object on one side of a picture gives the unpleasant impression that the whole picture is tilting toward that side; something is needed on the opposite side to provide a counterbalance. On the other hand, a perfectly balanced picture lacks excitement.

In some situations, an exact balance can add value because we delight in symmetry (Adkins & Norman, 2016; Bertamini et al., 2013). This appreciation is apparently shared with animals (Bertamini et al., 2018). One possible explanation is related to mate choice. All vertebrates have bilateral bodies, meaning they are, as a rule of thumb, meant to be symmetrical around a central plane. Breaches of this symmetry is a sign of inferior health and gene quality, which is why people tend to prefer a symmetrical partner. In design features, it may also be a question of reducing image complexity and making the visual impression more fluent (Chen et al., 2011). The positive aesthetic value of symmetry is perhaps best recognized in architecture, many buildings considered great works of art have symmetrical features.

### Functionality

A preference for symmetry can be seen as an example of a somewhat broader aesthetic element – that of functionality. The German philosopher Alexander Baumgarten, who first coined the term aesthetics back in the eighteenth century, considered beauty to rely on perfection of sensible information. Art should try to improve on nature. The idea is reflected in the more recent tradition referred to as the functionalist movement (Hansson, 2005). A central doctrine is that form should follow function.

It seems reasonable that looking for functionality and perfection relates to an agreeable sensation. For example, consuming sick animals or spoiled fruit may be dangerous, whereas eating the best specimens ought to keep you healthy; and it is important to recognize the features of a tool that make it useful. It therefore makes sense that the brain rewards us for noticing objects that have the right quality, the pleasant sensation stimulates us to distinguish good from bad and to obtain what is good.

In the case of art, function is not that obvious, but the principle is presumably reflected in that there should be perfection in the effort laid down by the artist. The relevance of this principle is exemplified by the fact that a minor fault in an otherwise great work of art drastically decreases its value. The fault could be due to the artist’s lack of concentration or later damage, such as a tear in the canvas.

Our evaluation of human appearance may be used to further illustrate the point. Experiments where people are asked to choose among a wide variety of photographic portraits indicate that the visually most attractive persons not only have symmetrical features, but also display average features (Grammer & Thornhill, 1994). In fact, the positive effect of symmetry may reflect that asymmetry breaches the rule of a preference for average features (Baudouin & Tiberghien, 2004). As a rule of thumb, a regular and prototypical anatomy implies health, while abnormalities can be interpreted as a sign of bad genes or a sick body.

### Depth and Movement

The eyes of primates are facing more forward compared to those of other mammals. Having both eyes pointing in the same direction limits the field of vision but allows for a better perception of depth. The primate design is presumably connected with the requirements of living in trees, where moving from branch to branch demands an ability to measure distances accurately. However, the ability to gauge depth requires practice. Practice implies reward-driven behavior. Thus, humans are presumably rewarded when they engage in the processing of relevant visual input. A typical criticism of amateur paintings is that they appear flat. We appreciate a painter who manages to create an impression of depth, thereby training an important processing skill.

A similar argument can be made regarding our capacity to detect movement. It is important for both humans and animals to take an interest in, and anticipate the trajectory of, moving objects because they could imply danger in the form of predators or opportunities in the form of prey. Thus, the brain should deliver rewards for relevant practice. Dance and films obviously involve movement, and sculptures occasionally include moveable components. True movement is not practical in paintings, but a picture may contain ‘lines’ that the eyes are induced to follow. In a culture where people are trained to read from left to right, lines running from the upper left toward the lower right are perceived as going down – and thus tend to give a depressive effect – while lines running along the opposite diagonal can be interpreted as elevating. The viewer who lets the eyes follow these lines can hope for drops of rewards – regardless of the direction – as they engage the capacity to investigate movement.

### The Human Factor

Our interest and curiosity for fellow humans, particularly the nature of their personality, suggest that we can appreciate man-made objects for what they tell about the creator. It means that we look for the ‘human touch’ and receive drops of rewards when we believe we find something.

Not only are we naturally nosy, but we are also rather fond of people. To associate with others diminishes negative sensations (loneliness) and adds agreeable feelings (geniality). An object of art contains clues regarding the mentality of the artist, and by viewing them we may sense a form of companionship with him or her.

A paradox in aesthetic theory is why people seem to enjoy images that are expected to evoke sadness or related negative feelings. A characteristic example is that many people appreciate films that make them cry. A painting depicting war and destruction, perhaps dominated by brown and dark colors, can be highly acclaimed. As mentioned before, even grief can feel good (O’Connor et al., 2008). The observation may be partly due to the idea that grief can be a desirable state for the genes in that the reaction solicits sympathy and help. The bystander can obtain brain rewards for empathic engagement with the victim, for example, people appreciate the chance to offer mental sympathy to the poor person depicted on the canvas, as well as to the artist who appears to be in such a miserable state.

Our capacity for compassion reflects our strong socializing instincts. By helping others, we enhance bonding; moreover, we are likely to receive something in return in the form of direct or indirect reciprocity. As in the case of the other examples of aesthetic elements, the sentiment does not need to be consciously recognized to have an impact on mood.

## Discussion

We suggest that the human inclination to add aesthetic elements to works of art, or other objects, is a consequence of how our brains are designed to influence behavior. The brain offers rewards when we focus our attention on features of nature that have certain visual qualities. We believe both the artist and the spectator may benefit from an awareness of relevant visual signals, as this knowledge should help them tune in to their aesthetic propensities. We propose a list of such features, referred to as aesthetic elements, that works independently of figurative and associative content. Alone, or in combinations, they add some extra pleasure to the viewing of works of art or other objects.

Rewards are initiated in the unconscious mind. They can influence your mood, and appraisal of art, even if the individual drops of rewards are not recognized, and even if conscious consideration tells you that what you are looking at is simply a blot of red paint. The reward of seeing a single aesthetic element can be minuscule, but in a successful piece of art the rewards combine to form a substantial delight.

The human brain is arguably the most flexible and adaptive of any brain, thus aesthetic appreciation will be drastically influenced by personal and cultural factors. This is obvious in the case of figurative aspects of art, but the impact of aesthetic elements will also vary. For example, those who enjoy riding a roller coaster where they experience the horror of falling, may also enjoy the thrill of an extremely unbalanced picture; while those who dislike roller coasters prefer the more balanced version. It is interesting to note that while art and design is largely a matter of personal and cultural taste, there is a higher concordance when evaluating the beauty of natural motifs (Vessel et al., 2018). The observation presumably reflects that all aesthetic elements have their origin in nature.

The present theory of aesthetic appreciation does not restrict the artist to narrow rules. The text is meant to help us understand aesthetic behavior, not to suggest a recipe for the creation of a masterpiece. For the sake of curiosity, excitement, or to evoke specific emotions the aesthetic elements may be deliberately disregarded. Although a delight in reddish or bluish colors may constitute an element of aesthetics, the artist may choose to use brown if the intention is to evoke sorrow rather than cheerfulness.

A particular visual input can induce pleasure, but it can also produce negative feelings. If the object of focus is considered as pleasing, it means that the sum of rewards and punishments is above zero. The best art may use aesthetic elements to enhance the figurative content. For example, the portrayal of a murder is typically rendered in dark brown, while a love scene is rendered in lush and warm colors. When painting a symmetrical house, you may choose to put it in the middle of the canvas, while a painting of someone climbing a rock could include highly unbalanced features to add drama.

The aesthetic elements are presumably pleasant partly due to the rewards offered for learning through play behavior. The eyes are arguably our most important sense organ, and we are encouraged to practice recognition and interpretation of visual stimuli, whether in the form of depth, symmetry, or finding the ‘human touch.’ The popularity of art may be partly due to humans carrying a considerable propensity for play behavior even as we age. Where most animals apparently use spare time simply to rest, we seek activities that offer rewards – including a visit to an art gallery. The choice of art as a diversion may have been boosted by sexual selection (Prum, 2012), but present cultural factors, including the monetary evaluation of objects of art, and the status obtained by being recognized as an artist, probably have a larger impact.

If one considers the pursuit of happiness as a suitable guiding principle for living (Grinde, 2012), taking and interest in aesthetics seems like an excellent strategy. Understanding why we do have the propensity to enjoy certain types of visual input should help in that pursuit.

## Data Availability

Data sharing not applicable to this article as no datasets were generated or analyzed during the current study.

## References

[CR1] Adkins OC, Norman JF (2016). The visual aesthetics of snowflakes. Perception.

[CR2] Bar M, Neta M (2006). Humans prefer curved visual objects. Psychological Science.

[CR3] Barrett D (2010). Supernormal stimuli: How primal urges overran their evolutionary purpose.

[CR4] Baudouin JY, Tiberghien G (2004). Symmetry, averageness, and feature size in the facial attractiveness of women. Acta Psychologica.

[CR5] Benton JR, DiYanni R (2012). Arts and culture: An introduction to the humanities.

[CR6] Berlyne DE (1970). Novelty, complexity, and hedonic value. Perception & Psychophysics.

[CR7] Berridge KC, Kringelbach ML (2015). Pleasure systems in the brain. Neuron.

[CR8] Bertamini M, Makin A, Rampone G (2013). Implicit association of symmetry with positive valence, high arousal and simplicity. i-Perception.

[CR9] Bertamini M, Silvanto J, Norcia AM, Makin AD, Wagemans J (2018). The neural basis of visual symmetry and its role in mid-and high-level visual processing. Annals of the New York Academy of Sciences.

[CR10] Birren F (2016). Color psychology and color therapy; a factual study of the influence of color on human life.

[CR11] Blood AJ, Zatorre RJ (2001). Intensely pleasurable responses to music correlate with activity in brain regions implicated in reward and emotion. Proceedings of National Academy of Science USA.

[CR12] Borgia GJSA (1986). Sexual selection in bowerbirds. Scientific American.

[CR13] Brielmann AA, Pelli DG (2018). Aesthetics. Current Biology.

[CR14] Cabanac M (1999). Emotion and phylogeny. Japanese Journal of Physiology.

[CR15] Camgöz N, Yener C, Güvenç D (2002). Effects of hue, saturation, and brightness on preference. Journal of Color Research.

[CR16] Casperson SL, Lanza L, Albajri E, Nasser JA (2019). Increasing chocolate’s sugar content enhances its psychoactive effects and intake. Nutrients.

[CR17] Cela-Conde CJ, Ayala FJ (2018). Art and brain coevolution. Progress in Brain Research.

[CR18] Chatterjee A, Vartanian O (2014). Neuroaesthetics. Trends in Cognitive Sciences.

[CR19] Chen CC, Wu JH, Wu CC (2011). Reduction of image complexity explains aesthetic preference for symmetry. Symmetry.

[CR20] Clottes J (2016). What is Paleolithic art?.

[CR21] Davies S (2012). The artful species: Aesthetics, art, and evolution.

[CR22] De Block A, Du Laing B (2010). Amusing ourselves to death? Superstimuli and the evolutionary social sciences. Philosophical Psychology.

[CR23] Dehaene S, Charles L, King J-R, Marti S (2014). Toward a computational theory of conscious processing. Current Opinion in Neurobiology.

[CR24] Dunbar RI (2001). What's in a baboon's behind?. Nature.

[CR25] Elliot AJ, Maier MA (2014). Color psychology: Effects of perceiving color on psychological functioning in humans. Annual Review of Psychology.

[CR26] Friedenberg J, Liby B (2016). Perceived beauty of random texture patterns: A preference for complexity. Acta Psychologica.

[CR27] Friston KJ, Price CJ (2011). Modules and brain mapping. Cognitive Neuropsychology.

[CR28] Gong R, Wang Q, Hai Y, Shao X (2017). Investigation on factors to influence color emotion and color preference responses. Optik.

[CR29] Graf, L. K., & Landwehr, J. R. (2017). Aesthetic pleasure versus aesthetic interest: The two routes to aesthetic liking. *Frontiers in Psychology, 8*. 10.3389/fpsyg.2017.00015 PMC5276863 28194119

[CR30] Grammer K, Thornhill R (1994). Human (Homo sapiens) facial attractiveness and sexual selection: The role of symmetry and averageness. Journal of Comparative Psychology.

[CR31] Grinde B (1996). The biology of visual aesthetics. Journal of Social and Evolutionary Systems.

[CR32] Grinde B (2012). The biology of happiness.

[CR33] Grinde B (2016). The evolution of Consciousenss.

[CR34] Grinde B (2018). Did consciousness first evolve in the amniotes?. Psychology of Consciousness: Theory, Research, and Practice.

[CR35] Grinde B, Patil GG (2009). Biophilia: Does visual contact with nature impact on health and well-being?. Int J Env Res PubHealth.

[CR36] Hansson SO (2005). Aesthetic functionalism. Contemporary aesthetics.

[CR37] Hekkert P, Snelders D, Van Wieringen PC (2003). ‘Most advanced, yet acceptable’: Typicality and novelty as joint predictors of aesthetic preference in industrial design. British Journal of Psychology.

[CR38] Hodgskiss, T. (2020). Ochre use in the middle stone age. *Oxford Research Encyclopedia of Anthropology, 1*. 10.1093/acrefore/9780190854584.013.51.

[CR39] Hurlbert AC, Ling Y (2007). Biological components of sex differences in color preference. Current Biology.

[CR40] Kissel M (2018). Non-modern humans were more complex—And artistic—Than we thought. Anthropology Newsletter.

[CR41] Konecni VJ (2003). The golden section: Elusive, but detectable. Creativity Research Journal.

[CR42] Kozbelt A (2017). Tensions in naturalistic, evolutionary explanations of aesthetic reception and production. New Ideas in Psychology.

[CR43] Lacey, S., Hagtvedt, H., Patrick, V. M., Anderson, A., Stilla, R., Deshpande, G., ... Sathian, K. (2011). Art for reward’s sake: Visual art recruits the ventral striatum. *NeuroImage, 55*(1), 420–433. 10.1016/j.neuroimage.2010.11.027 PMC3031763 21111833

[CR44] Landwehr JR, Labroo AA, Herrmann A (2011). Gut liking for the ordinary: Incorporating design fluency improves automobile sales forecasts. Marketing Science.

[CR45] Leknes S, Tracey I (2008). A common neurobiology for pain and pleasure. Nature Reviews Neuroscience.

[CR46] Lewis S, South A (2012). The evolution of animal nuptial gifts. Advances in the Study of Behavior.

[CR47] Lieberman MD, Eisenberger NI (2009). Neuroscience pains and pleasures of social life. Science.

[CR48] Lipowski ZJ (1974). Sensory overloads, information overloads and behavior. Psychotherapy and Psychosomatics.

[CR49] Matthen M (2017). The pleasure of art. Australasian Philosophical Review.

[CR50] Menninghaus W, Wagner V, Wassiliwizky E, Schindler I, Hanich J, Jacobsen T, Koelsch S (2019). What are aesthetic emotions?. Psychological Review.

[CR51] Morris D (1994). The human animal.

[CR52] O'Connor MF, Wellisch DK, Stanton AL, Eisenberger NI, Irwin MR, Lieberman MD (2008). Craving love? Enduring grief activates brain's reward center. NeuroImage.

[CR53] Palmer SE, Schloss KB (2010). An ecological valence theory of human color preference. Proceedings of the National Academy of Sciences.

[CR54] Papadimitriou F (2020). Spatial complexity, visual complexity and aesthetics. Spatial complexity.

[CR55] Pjrek, E., Friedrich, M.-E., Cambioli, L., Dold, M., Jäger, F., Komorowski, A., ... Winkler, D. (2020). The efficacy of light therapy in the treatment of seasonal affective disorder: A meta-analysis of randomized controlled trials. *Psychotherapy and Psychosomatics, 89*(1), 17–24. 10.1159/000502891 31574513

[CR56] Prum RO (2012). Aesthetic evolution by mate choice: Darwin's really dangerous idea. Philosophical Transactions of the Royal Society B: Biological Sciences.

[CR57] Rolls ET (2017). Neurobiological foundations of aesthetics and art. New Ideas in Psychology.

[CR58] Schindler I, Hosoya G, Menninghaus W, Beermann U, Wagner V, Eid M, Scherer KR (2017). Measuring aesthetic emotions: A review of the literature and a new assessment tool. PLoS One.

[CR59] Schlebusch, C. M., Malmström, H., Günther, T., Sjödin, P., Coutinho, A., Edlund, H., ... Soodyall, H. (2017). Southern African ancient genomes estimate modern human divergence to 350,000 to 260,000 years ago. *Science, 358*(6363), 652–655. 10.1126/science.aao6266 28971970

[CR60] Skov M, Kringelbach M, Berridge K (2010). The pleasure of art. Pleasures of the brain.

[CR61] Skov M, Nadal M (2020). A farewell to art: Aesthetics as a topic in psychology and neuroscience. Perspectives on Psychological Science.

[CR62] Tamietto M, de Gelder B (2010). Neural bases of the non-conscious perception of emotional signals. Nature Reviews Neuroscience.

[CR63] Tinio PP, Gartus A (2018). Characterizing the emotional response to art beyond pleasure: Correspondence between the emotional characteristics of artworks and viewers’ emotional responses. Progress in Brain Research.

[CR64] Torres A, Serra J, Llopis J, Delcampo A (2020). Color preference cool versus warm in nursing homes depends on the expected activity for interior spaces. Frontiers of Architectural Research.

[CR65] Van der Berg S (2020). Aesthetic hedonism and its critics. Philosophy Compass.

[CR66] Vartanian O, Skov M, Vartanian O (2018). Conscious experience of pleasure in art. Neuroaesthetics.

[CR67] Vessel EA, Maurer N, Denker AH, Starr GG (2018). Stronger shared taste for natural aesthetic domains than for artifacts of human culture. Cognition.

[CR68] Westphal-Fitch G, Fitch WT (2018). Bioaesthetics: The evolution of aesthetic cognition in humans and other animals. Progress in Brain Research.

[CR69] Wright JS, Panksepp J (2012). An evolutionary framework to understand foraging, wanting, and desire: The neuropsychology of the SEEKING system. Neuropsychoanalysis.

[CR70] Xu HA, Modirshanechi A, Lehmann MP, Gerstner W, Herzog MH (2021). Novelty is not surprise: Human exploratory and adaptive behavior in sequential decision-making. PLoS Computational Biology.

